# Multiscale analysis of mechanical behavior of multilayer steel structures fabricated by wire and arc additive manufacturing

**DOI:** 10.1080/14686996.2020.1788908

**Published:** 2020-07-22

**Authors:** Ikumu Watanabe, Zhengzhong Sun, Houichi Kitano, Kenta Goto

**Affiliations:** a Research Center for Structural Materials, National Institute for Materials Science, Tsukuba, Ibaraki, Japan; b Graduate School of Pure and Applied Sciences, University of Tsukuba, Tsukuba,Ibaraki, Japan

**Keywords:** Wire and arc additive manufacturing, stainless steel, finite element method, instrumented indentation, multiscale characterization, 106 Metallic materials, 305 Plasma / Laser processing, 400 Modeling/Simulations, 500 Characterization

## Abstract

The mechanical behavior of multilayer steel structures fabricated via wire and arc additive manufacturing (WAAM) has been investigated from the multiscale perspective. The multimaterial WAAM approach can control a heterogeneous structure and improve its mechanical properties. In this study, WAAM equipment based on plasma arc welding was used to fabricate two pairs of single- and duplex-phase multilayer steel structures using austenitic and martensitic stainless steel wires. The heterogeneity of these structures was characterized through micro-indentation tests. In addition, tensile tests of the multilayer structures were conducted to evaluate the effect of heterogeneity on macroscopic material properties. The deformation behavior of the heterogeneous multilayer steel structures was investigated by comparison with the finite element simulations of tensile tests in which the finite element models were created according to the estimated local elastoplastic properties from the results of micro-indentation tests. The micro-indentation tests revealed that the local mechanical properties significantly change during WAAM in cases where martensitic stainless steel wire was used. Additionally, strain-induced transformation plasticity was particularly observed in duplex cases, caused by the metastable austenite phase formed according to the thermal history and through the mixing of alloy elements. Thus, the heterogeneity of the multilayer steel structures became more complicated than its design, and consequently, its macroscopic mechanical properties exceeded the upper and lower bounds of a micromechanic estimation. The results show the potential to fabricate a structure having a unique mechanical behavior via the multimaterial WAAM approach.

## GRAPTICAL ABSTRACT

**Figure:**
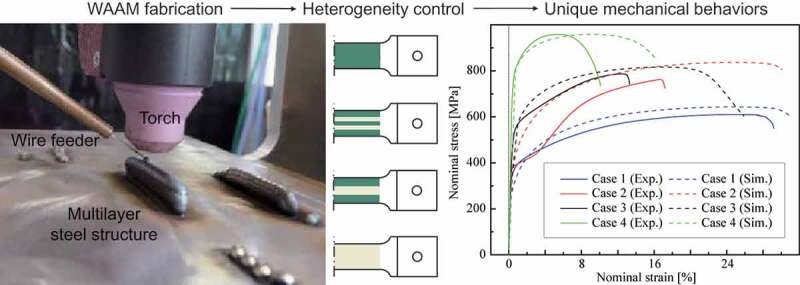

## 1. Introduction

The trade-off between strength and ductility is a common dilemma in structural metals. Heterogeneous structure design has been recognized as an effective approach to overcome this dilemma as it avoids the localization of stress or strain [1,2]. In this context, various metallic materials containing a heterogeneous structure have been developed such as bimodal grain size structure [3,4], ultrafine fibrous grain structure [5], harmonic structure [6–8], Van Gogh’s sky structure [9,10], and the multilayer structure [11]. Bimodal grain-size structure [3,4] and ultrafine fibrous grain structure [5] were fabricated by severe plastic deformation processes; hence, the size and shape are limited. Although harmonic structure [6–8] which is shell-core bimodal grain size structure is capable of near-net-shape manufacturing using powder metallurgy, it is difficult to fabricate big components. Van Gogh’s sky structure [9,10] based on segregation of alloy element appears in only a special alloy system; therefore, the applicability is very limited. The multilayer steels [11] were fabricated via roll-bonding and successfully improved the combination of strength and ductility compared with conventional steels by independently allocating the soft and hard phases along the thickness direction. The partitioned stresses in these soft and hard phases improve the multilayer steels’ ductility. Ojima et al. [12] validated the deformation mechanism of multilayer steels through neutron diffraction measurement, wherein the stress not only distributed soft phases but also hard phases effectively during the uniaxial tensile test. This multilayer concept has been extended to various combinations such as the Mg–steel multilayer sheet [13–15].

According to these past studies, multiphase or multiconstituent metals improve the strength–ductility relationship via the heterogeneous distribution of stress or strain. The next step is controlling the heterogeneity to maximize the improvement of mechanical properties. Classical micromechanics [16–18] describe the upper and lower bounds in the relationship between elastic stiffness and volume fraction of a duplex elastic solid, implying that the topology of a phase affects the stiffness of the composite. Watanabe et al. [19] stated that these upper and lower bounds are experimentally applicable in the estimation of yield strength of duplex constituent steels; furthermore, they developed a computational design approach comprising a morphology of duplex microstructures to maximize the bulk yield strength. Matsuno et al. [20] also discussed the morphological effect of the martensite phase in dual-phase steel on tensile strength. Currently, the macroscopic material behavior described by a tensorial relationship between stress and strain has been characterized from a microscopic heterogeneity via numerical material testing based on the finite element method for the representative volume element [21,22] and designed by combining an inverse analysis method with numerical material testing. However, fabrication approaches for the computationally designed heterogeneous structure have been very limited in the present state.

Additive manufacturing (AM), i.e. three-dimensional (3D) printing, has lately gained considerable attention as a fabrication process for designed structures [23]. This approach has been applied to various materials, from polymers to metals and ceramics, and further extended to multimaterials [24,25]. For the fabrication of large structural parts, direct energy deposition is a promising approach. In this study, we use wire and arc additive manufacturing (WAAM), one of direct energy deposition processes, owing to the following favorable features [26–29].

• Wire is easier to handle than powder for multimaterial AM.

• The deposition rate of WAAM is higher than that of other AM approaches.

• WAAM can produce fully dense metal products.

• The mechanical properties of the product are similar to those of forging [30] or powder metallurgy [31] if the process is successful.

However, severe residual stress and distortion are observed in a fabricated product and its substrate because of the high heat input on one side of the substrate. Additionally, mechanical anisotropy in the horizontal and vertical directions is inevitable in the product. Despite these issues, WAAM is a promising approach to fabricate multimaterial structures.

In this study, we investigated the multiscale material behavior of multilayer steel structures fabricated via WAAM in an application of the multimaterial AM. Specifically, macro- and microscopic mechanical tests were conducted and metallographic microstructures were observed in the multimaterial multilayer structures. The effect of heterogeneity was analyzed based on the computational simulations of the tensile tests performed using finite element models of the multimaterial multilayer structures while incorporating the experimental data.

## 2. Fabrication of multilayer steel structures

**Figure 1.**
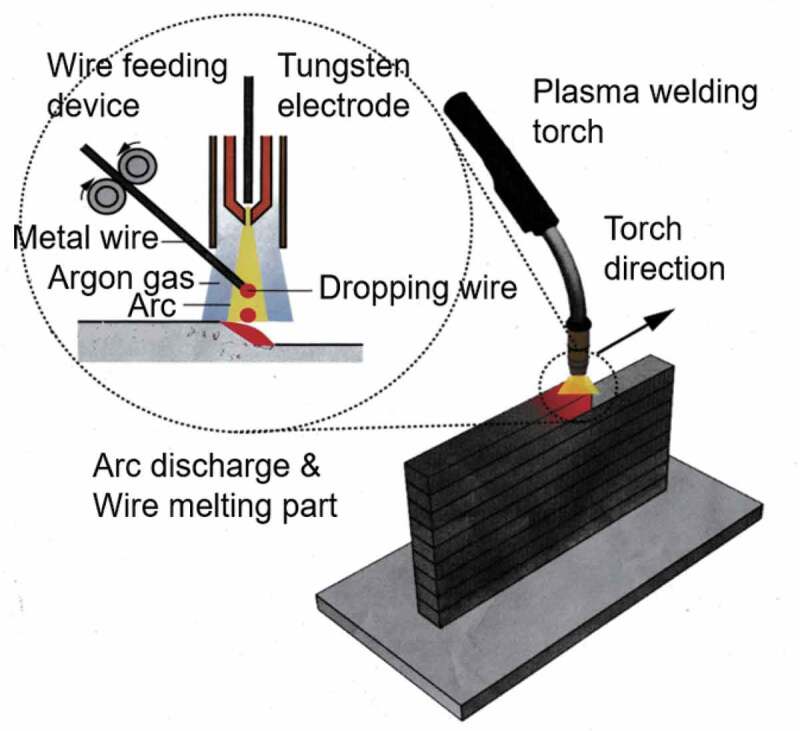
Schematic of wire and arc additive manufacturing (WAAM) equipment based on plasma arc welding.

In this study, our original WAAM equipment based on plasma arc welding was used to fabricate multilayer steel structures, as illustrated in Figure 1. This equipment is designed to fabricate smaller specimens than standard WAAM equipment using welding machines, including the electron-beam machine and the cold metal transfer machine, and to facilitate changing of wires.

**Figure 2.**
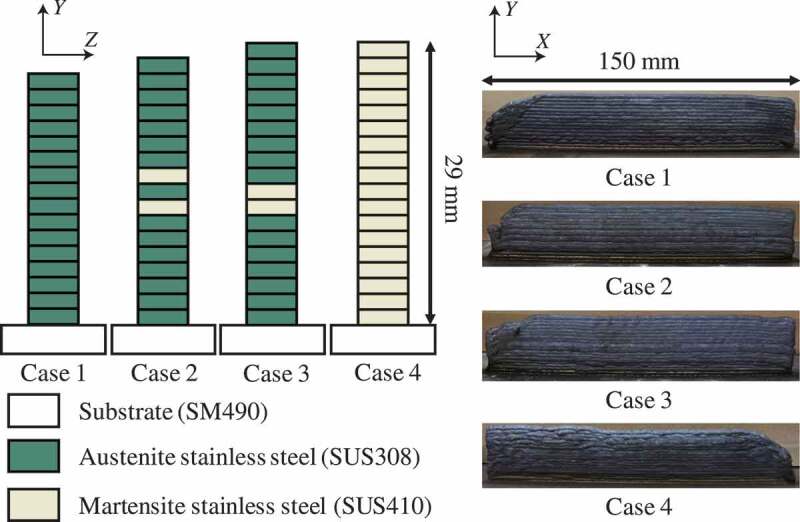
Multilayer steel structures fabricated via WAAM.

Two commercial welding wires of austenitic stainless steel (SUS308) and martensitic stainless steel (SUS410) having a diameter of 0.6 mm each were chosen as the raw materials of the multilayer steel structures. Four types of multilayer steel structures were fabricated on a substrate composed of carbon steel (SM490), as shown in Figure 2. Table 1 lists the alloy compositions of SM490, SUS308, and SUS410. These structures have two types of heterogeneity stemming from their layered structure and duplex phases. The structures of cases 1 and 4 were designed as single-phase multilayer structures composed of full austenitic and martensitic stainless steel, respectively. In contrast, the structures of cases 2 and 3 were designed as duplex-phase multilayer structures containing two martensite layers in austenite layers, as shown in Figure 2. In case 2, one austenite layer was put between the two martensite layers; on the other hand, two martensite layers were put consecutively in case 3. In WAAM, the torch’s travel speed and wire feed speed were defined as 60 and 1200 mm/min, respectively. Notably, setting the currents of the arc plasma for both wires while considering the difference in thermal conductivity between the substrate and the fabricated layers is necessary. This process parameter was optimized for each wire, as shown in Table 2.

**Table 1.** Alloy compositions of SM490, SUS308, and SUS410 [wt%].

Type	C	Si	Mn	P	S	Ni	Cr	Mo
SM490	≤0.20	≤0.55	≤1.65	≤0.035	≤0.035	–	–	–
SUS308	≤0.08	≤0.65	1.0-2.5	≤0.03	≤0.03	9.0-11.0	19.5-22.0	≤0.75
SUS410	≤0.12	≤0.5	≤0.6	≤0,03	≤0.03	≤0.6	11.5-13.5	≤0.75

**Table 2.** Optimized current for the fabrication process.

Wire	SUS308			SUS410		
Layer number	1st	2nd	≥3rd	1st	2nd	≥3rd
Current [A]	50	40	35	60	45	40

The height of one layer was approximately 1.5 mm. Hence, the total height of these specimens was less than 30 mm.

## 3. Heterogeneity of multilayer steel structures

In this section, the investigation conducted on the heterogeneity of the multilayer steel structures fabricated via WAAM using micro-indentation tests is outlined. Micro-indentation tests were performed by a dynamic micro-indentation machine (Shimadzu Co., Japan) from near the substrate to the top of the multilayer structure at intervals of 0.5 mm (about one-third of the one-layer height) to characterize the distribution of local mechanical properties in the four multilayer steel structures. Here, the maximum applied load was 1,960 mN using a standard Berkovich indenter.

### 3.1. Hardness distributions

**Figure 3.**
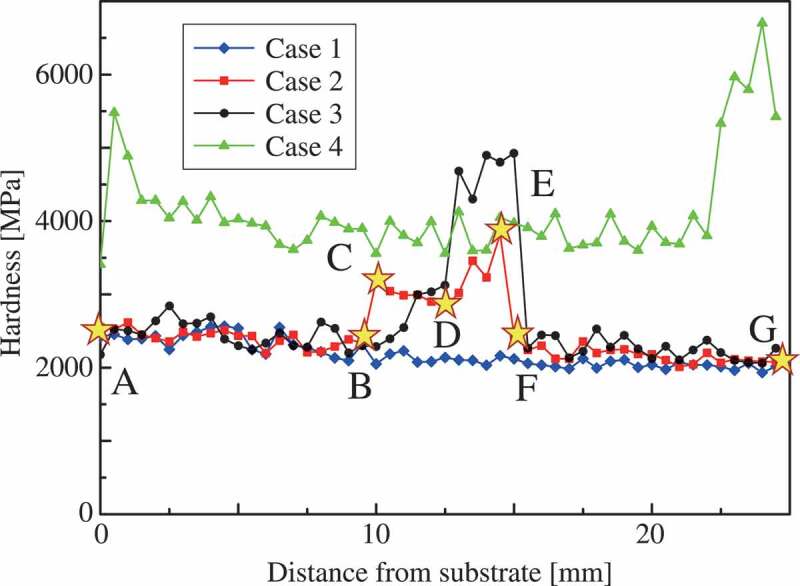
Indentation hardness distributions of multilayer steel structures.

The indentation hardness distributions of four cases are depicted in Figure 3. Indentation hardness [32] was calculated from the load–displacement curve of a micro-indentation test through the Oliver–Pharr method [33]. The hardness of the martensite phase is typically higher than that of the austenite phase. In addition, the martensite phase is more sensitive in terms of the effect of thermal history on its strength and underlying microstructure. Therefore, in case 4, the hardness of the structure near the substrate and top area was higher than those of other areas because of different thermal histories. In cases 1 and 4 of the single-phase multilayer structures, a variation in hardness can be found around the middle of the structures; however, this variation is not major in comparison to the difference between martensite and austenite phases. In cases 2 and 3 of duplex-phase multilayer structures, the hardness around the martensite area was higher than that of other areas. Note that higher hardness was measured around the austenite layer located between two martensite layers in case 2.

**Figure 4.**
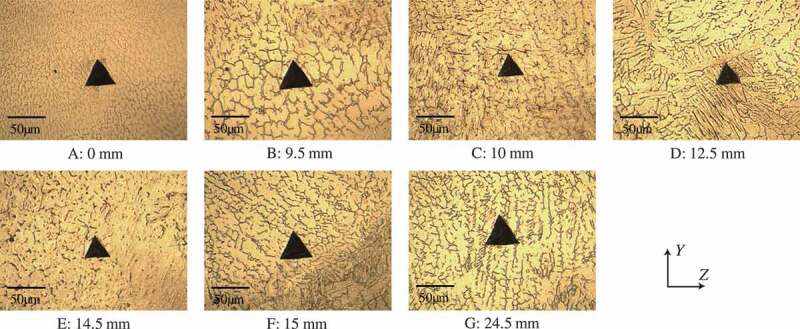
Microstructure around indentation areas in Case 2.

Figure 4 shows the optical micrographs around the indentation mark at seven representative points in case 2. In Figure 4, the microstructures around the martensite layers, from C to F of Figure 3, over 5 mm length, contain both martensite and austenite phases. The size of the indentation mark is approximately 40 μm, which is enough big to characterize the local mechanical properties affected by the heterogeneous microstructure.

**Figure 5.**
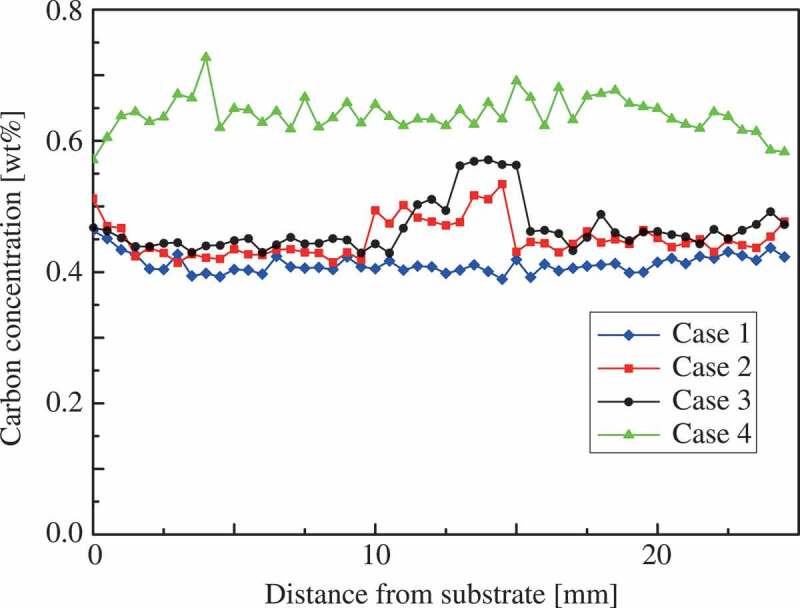
Carbon distributions of multilayer steel structures.

Figure 5 shows the distributions of carbon content observed using an electron probe micro-analyzer (EPMA) (JEOL Ltd., Japan) at the side of the indentation marks in all tested multilayer steel structures. These data are qualitative in nature owing to the measurement accuracy of EPMA for carbon; however, these data correspond to the hardness distributions shown in Figure 3 and then the actual position of martensite layers can be confirmed in cases 2 and 3 from Figure 5. Note that the actual position is possibly the height of one layer away from the designed position shown in Figure 2 in the middle of the structures because of the difference in the process conditions. As shown in Table 1, the martensite phase contains one and a half times as much carbon as the austenite phase. Figure 5 shows that, in the duplex-phase structures, the carbon distributes wider compared with the design of Figure 2 because the carbon element diffused from the martensite phase to the austenite phase during WAAM. As a result of mixture alloy components and thermal history, the dual-phase steel microstructure appeared in cases 2 and 3. Notably, the larger area of the martensite phase is possibly observed on the surface than the inside because strain-induced martensitic transformation occurs during sample preparation. We discuss this topic of the strain-induced martensitic transformation in the next section.

### 3.2. Pile-up height distribution

**Figure 6.**
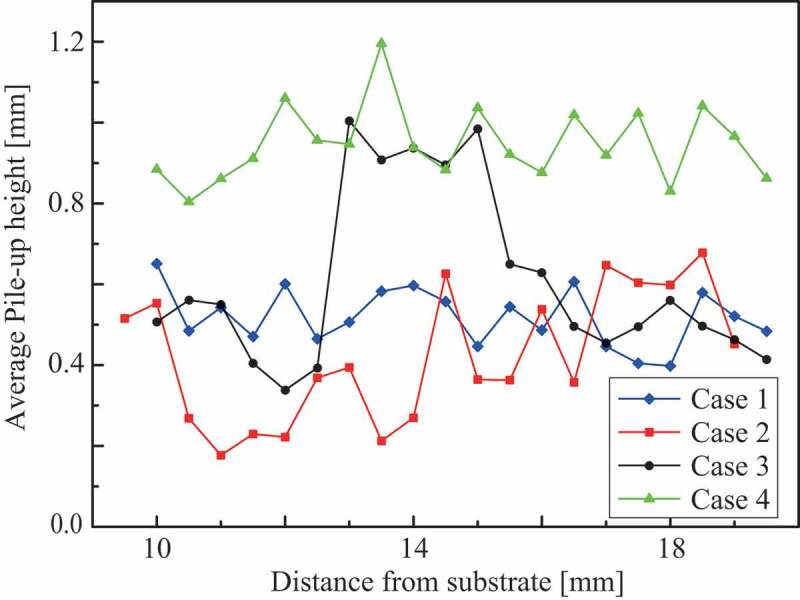
Pile-up height distributions of multilayer steel structures.

Stress–strain curves on each indentation test were estimated through the single indentation estimation approach based on the load–displacement curve and pile-up height [34,35]. The pile-up height relates to plastic strain-hardening [36,37]. Following an earlier study [34], the average pile-up height along three lines passing through a vertex and the midpoint of the opposite side were used to estimate the plastic properties, where the pile-up height was measured using a confocal laser microscope (Lasertec Co., Japan). The distributions of pile-up height around the middle of the layer structures are depicted in Figure 6. These distributions exhibit a clear difference between the martensite phase, austenite phase, and the mixture area. The estimated stress–strain curves in the multilayer steel structures were used to build finite element models of tensile specimens in the next section.

## 4. Tensile tests of multilayer steel structures

Tensile tests of the multilayer steel structures fabricated via WAAM were conducted to investigate the effect of heterogeneity on macroscopic mechanical behaviors. In addition, finite element simulations of the tensile tests were performed.

### 4.1. Test specimens and their finite element simulations

**Figure 7.**
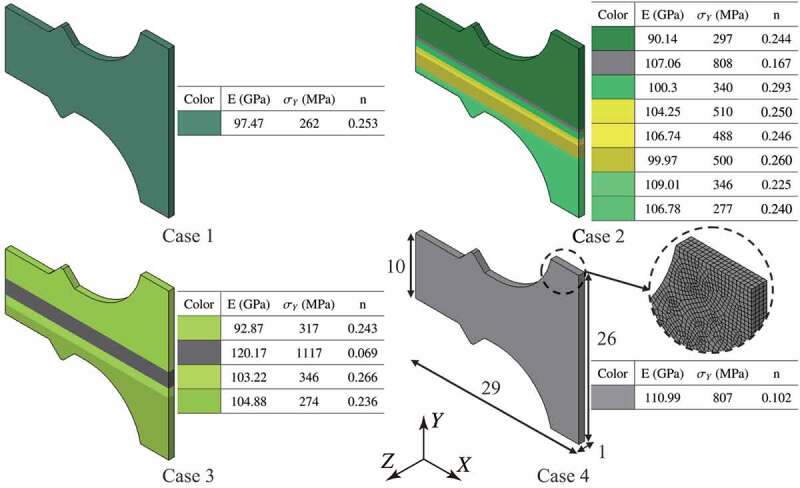
Finite element models of multilayer steel structures for the tensile test. The material constants were estimated from results of micro-indentation tests.

Test specimens and their finite element models were prepared for the tensile tests, as depicted in Figure 7, from the center of the multilayer steel structures, i.e. the duplex-phase area in cases 2 and 3. The mound part of the test specimen was designed to measure displacement through a contact-type extensometer. In the tensile test, an uniaxial deformation was imposed in the direction parallel to the layers.

In finite element simulations, a standard quasi-static initial boundary value problem of elastoplastic deformation at finite strain was solved using the Lagrangian mesh and an implicit calculation scheme, in which the body force caused by gravity was ignored on account of its small effect in the tensile tests. For the constitutive model, isotropic hypoelasticity and metal plasticity were employed because it is necessary to use such a simple constitutive model in order to estimate the material parameters based on single indentation [34,35].

The finite element model was discretized by 28,800 8-node hexahedron finite elements, where mirror symmetry was implemented at the center of the test specimens. In Figure 7, the estimated material parameters of Young’s modulus *E*, yield strength $\sigma_{y}$, and plastic strain-hardening exponent *n* were applied, with Poisson’s ratio predefined as 0.3. Similar values of these material parameters were merged into one group, and then finite element models of cases 1 and 4 were found to be homogeneous and those of cases 2 and 3 were determined to be multilayer structures composed of eight and four groups, respectively. Note that the finite element models were built on the basis of the experimental data of micro-indentations without any fitting for the tensile tests. Consequently, the heterogeneous structures of cases 2 and 3 are different from those shown in Figure 2.

### 4.2. Results and discussion

**Figure 8.**
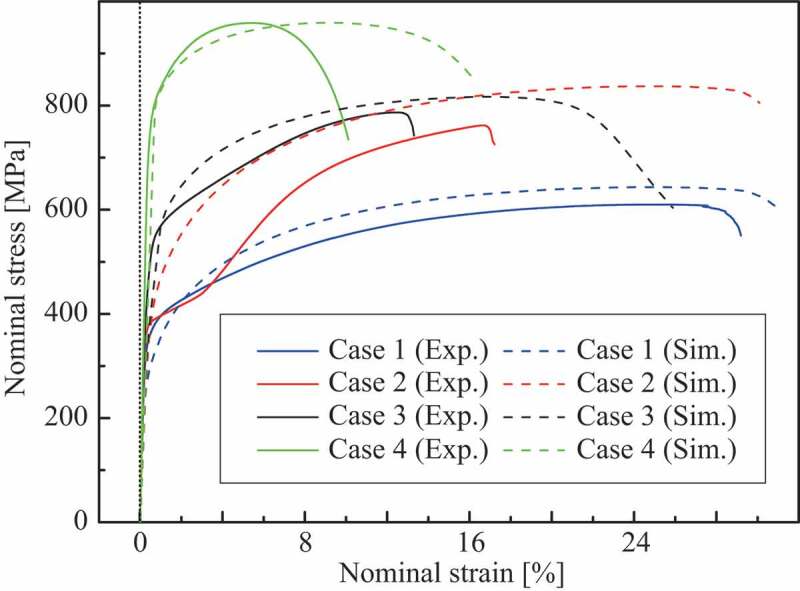
Stress–strain curves of multilayer steel structures.

Experiments and simulated stress–strain curves are depicted in Figure 8, wherein the material responses of finite element simulations are represented using a dashed line. Yield strength at 0.2% offset, tensile strength, and elongation as the macroscopic mechanical properties are summarized in Table 3, where the tensile strength and elongation are defined as true axial stress and strain at the point of maximum nominal stress value in the stress–strain curve following an earlier study by Matsuno et al. [20]. The residual error is defined as

(1) $$Error=\frac{x_{\exp}-x_{sim}}{x_{sim}},$$

**Table 3.** Macroscopic mechanical properties of multilayer steel structures.

		Yield strength [MPa]	Tensile strength [MPa]	Elongation [%]
Case 1	Exp.	355.7	763.1	22.4
	Sim.	300.6	803.3	22.2
	Error	−0.1549 [–]	0.0527 [–]	−0.01 [–]
Case 2	Exp.	378.8	888.9	15.4
	Sim.	404.8	1044.3	22.1
	Error	0.0686 [–]	0.1748 [–]	0.43 [–]
Case 3	Exp.	494.0	884.7	11.7
	Sim.	443.9	955.1	15.6
	Error	−0.1014 [–]	0.0796 [–]	0.33 [–]
Case 4	Exp.	780.6	1009.8	5.3
	Sim.	826.7	1046.9	8.8
	Error	0.0591 [–]	0.0367 [–]	0.66 [–]

where $x_{\exp}$ and $x_{sim}$ are values of experiment and simulation, respectively.

**Figure 9.**
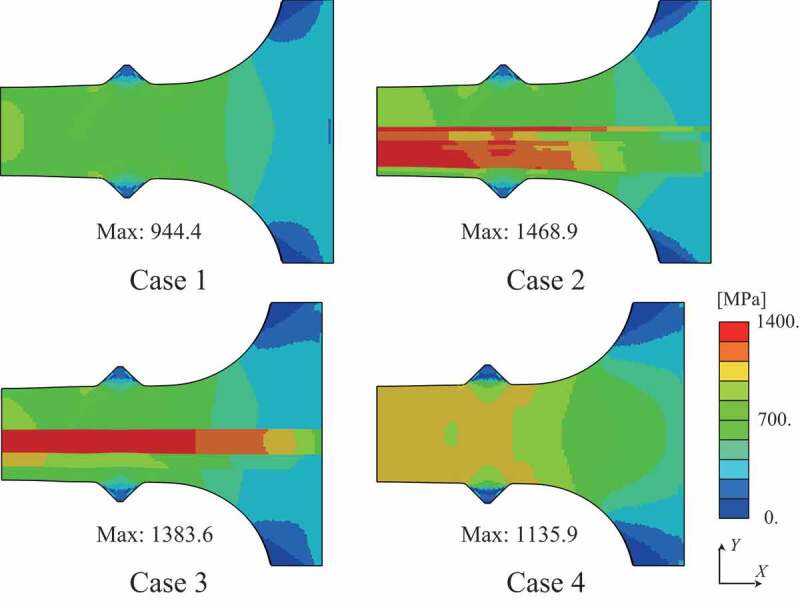
Distribution of von Mises stress at maximum stress state in the stress–strain curve.

**Figure 10.**
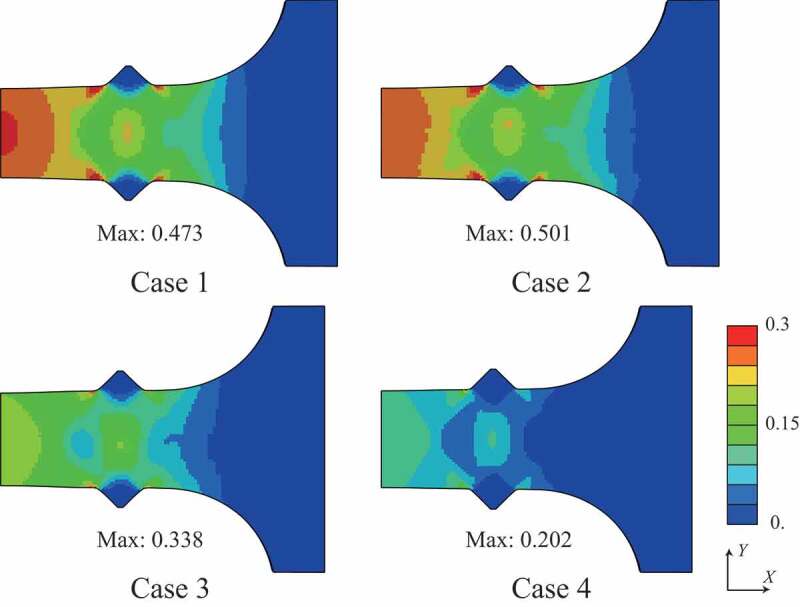
Distribution of equivalent plastic strain at maximum stress state in the stress–strain curve.

**Figure 11.**
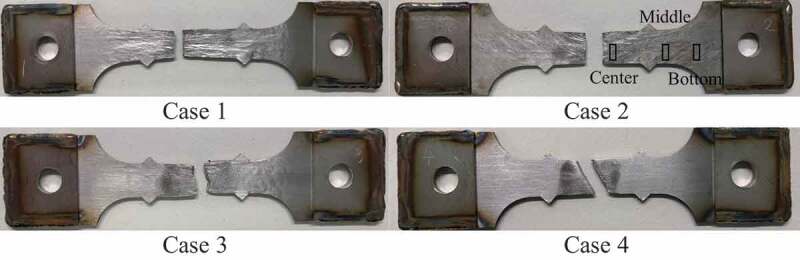
Test specimens after the tensile test.

The distributions of von Mises stress and equivalent plastic strain at the maximum nominal stress state are shown in Figures 9 and 10, respectively. The test specimens after the tensile tests are shown in Figure 11, which clearly shows that the deformation mode of case 4 differed from those of cases 1, 2, and 3 because of the higher strength of the martensite phase. Although the fracture behavior was not covered in this study because damage modeling was not employed, the stress–strain curves of the simulations agree with those of the experiments until plastic hardening, except for case 2. Using the simulation results, we can discuss qualitatively the macroscopic deformation behaviors.

Even though the structures of cases 2 and 3 contain the same amount of martensite phase, the stress–strain curves of cases 2 and 3 were different in both the experiments and simulations. Figures 9 and 10 present the stress concentrated in the duplex-phase area; however, the strain is continuously distributed in cases 2 and 3, caused by the imposed deformation in the direction parallel to the layers. The above boundary condition leads to a continuous strain state of the multilayer structures; therefore, the material responses were close to the upper bound. Based on an earlier study by Watanabe et al. [19], the upper and lower bounds of yield strength were calculated from the single-phase yield strength of cases 1 and 4 as 440.7 MPa and 399.2 MPa, respectively, with the volume fraction of martensite phase assumed as 20% from the original design. Note that the experimental results of cases 2 and 3 exceeded these bounds.

**Figure 12.**
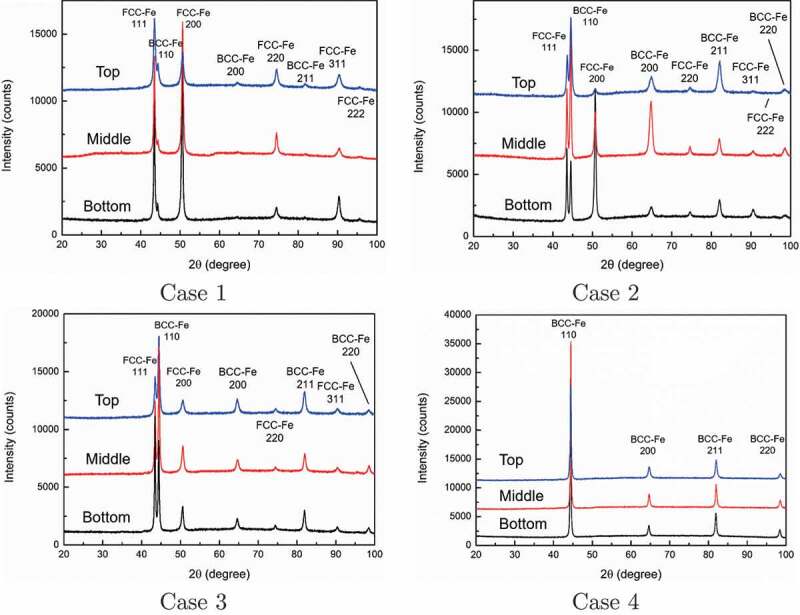
Crystal structure analysis using X-ray diffraction at three areas of test specimens after the tensile test.

The material responses of multilayer steels are unique two-stage stress–strain curves as shown in Figure 8. Especially that of case 2 is significant, which was confirmed duplicability. Such a two-stage stress–strain curve is typically observed in transformation plasticity [38]. To confirm the strain-induced martensite transformation, the crystal structure was analyzed via X-ray diffraction (Rigaku Co., Japan) at three points of each specimen, as shown in Figure 11 of case 2. The measurement points correspond to the different extents of deformation, as shown in Figure 10. In the specimen, the material at the center area close to the fracture surface deformed more than the other area. In contrast, the extent of deformation in the bottom area is smaller because of the wider cross-sectional area. The results of crystal structure analysis using X-ray diffraction are depicted in Figure 12. Also, Table 4 summarizes the analysis results as the mass fraction of the face-centered cubic (FCC) structure corresponding to austenite phase and body-centered cubic (BCC) structure corresponding to the martensite phase at the carbon content below 0.6 wt% [39]. In case 4, the mass fraction of martensite phase remained constant at 100% as designed, whereas in cases 1, 2, and 3, it increased with the increasing extent of deformation; i.e. strain-induced martensitic transformation occurred from a metastable austenite phase formed through WAAM, as mentioned in Section 3.1. In particular, the transformation is notable in cases 2 and 3 of the duplex-phase structures. Considering the carbon content of SUS308, the material of case 1 can transform. Furthermore, the increase in the carbon content around the area of the duplex structure in cases 2 and 3 accelerates the behavior. The two-stage stress–strain curve appeared in case 2 because the unstable area of case 2 is wider than that of case 3.

**Table 4.** Mass fraction of face-centered cubic (FCC)/body-centered cubic (BCC) structures of four cases in three measurement areas [%].

FCC/BCC	Bottom area	Middle area	Center area
Case 1	93.9/6.1	93.5/6.5	81.4/18.6
Case 2	61.0/39.0	38.7/61.3	34.4/65.6
Case 3	58.6/41.4	44.0/56.0	39.9/60.1
Case 4	0.0/100.0	0.0/100.0	0.0/100.0

## 5. Conclusion

In this study, the effect of multilayer steel structures fabricated via WAAM on the responses of macroscopic material was investigated using multiscale characterization approaches. In duplex-phase multilayer structures, the mechanical behavior of exceeding upper and lower bounds can be observed by forming a mixture alloy state. This multimaterial WAAM enables us to design new functional composite structures by another combination of wires and control of the process parameters. From the metallurgical aspect, thermal treatment is effective to improve the mechanical properties. In addition, the computational modeling framework bridging between processes, microstructure, and properties is required to design effectively such multiscale heterogeneous structures for its practical applications.
